# Therapeutic and prophylactic gastrectomy in a family with hereditary diffuse gastric cancer secondary to a CDH1 mutation: a case series

**DOI:** 10.1186/s12957-018-1415-5

**Published:** 2018-07-14

**Authors:** Olsi Gjyshi, Pankaj Vashi, Laura Seewald, Mitra Kohan, Elham Abboud, Eric Fowler, Revathi Suppiah, Hatem Halabi

**Affiliations:** 1 Presence Saint Joseph Hospital, Chicago, IL USA; 2 Cancer Treatment Centers of America, Zion, IL USA; 3 University of Michigan, Ann Arbor, MI USA; 4 Chicago Medical School, North Chicago, IL USA

**Keywords:** Hereditary diffuse gastric cancer (HDGC), *Cadherin-1* (*CDH1*), Lobular breast cancer (LBC)

## Abstract

**Background:**

Gastric cancer is the fifth most prevalent and the third most lethal cancer worldwide, causing approximately 720,000 deaths annually. Although most cases of gastric cancers are sporadic, one of its inherited forms, hereditary diffuse gastric cancer (HDGC), constitutes about 1–3% of cases. Interestingly, females in families with HDGC are also predisposed to developing lobular breast cancer (LBC). Recent analyses have identified loss-of-function germline mutations in *cadherein-1* (*CDH1*) as a culprit in HDGC and LBC. This discovery fueled several sequencing analyses and case series reports analyzing the pattern of inheritance of *CDH1* and its propensity to induce HDGC. In 2015, a multinational and multidisciplinary task force updated the guidelines and criteria for screening, diagnosing, and managing HDGC.

**Case presentation:**

Here, we present a case series of three siblings with family history of HDGC who tested positive for the *CDH1* mutation and describe their surgical treatment course, post-operative management, and follow-up as they pertain to the updated guidelines.

**Conclusions:**

Despite recent updates in guidelines in the diagnosis and management of HDGC, the disease remains challenging to address with patients given the high level of uncertainty and the comorbidities associated with prophylactic intervention. We strongly recommend that an interdisciplinary team inclusive of clinical and surgical oncologists, along with geneticists, social work, and psychological support, should follow the patients in a longitudinal and comprehensive manner in order to achieve full recovery and return to normalcy, as with our patients.

## Background

Cancer is gaining ground over cardiovascular disease as the leader of all-cause mortality, and gastric cancer is an important contributor with approximately 720,000 annual deaths [1, 2]. Although the majority of gastric cancers are sporadic in nature, approximately 1–3% of the reported cases are related to inherited cancer predisposition syndromes [3, 4], known as hereditary diffuse gastric cancer (HDGC). Several genetic mutations have been linked to HDGC, with heterozygous *cadherin-1* (*CDH1*) germline mutations having the strongest clinical association and being present in up to 40% of cases [4]. *CDH1* codes for the tumor suppressor protein E-cadherin and serves as an adhesion molecule at the basement membrane of epithelial cells and mediates downstream signaling cascades [5].

In families with *CDH1* mutations, there is a cumulative risk of 70 and 56% in males and females, respectively, of developing HDGC by age 80 [4]. Additionally, *CDH1* mutations are highly associated with development of lobular breast cancer (LBC), with a cumulative risk of 42% in females by age 80 [4]. These estimates demonstrate the significance of the *CDH1* germline mutations in developing HDGC and LBC, especially when considering that the lifetime risk of the general population in developing these cancers is 0.9% in either gender for diffuse gastric cancer (DGC) and 12% for breast cancer (BC) in females [2].

These observations, combined with advances in genetic diagnostics, endoscopic methods, and laparoscopic surgery, led a multinational expert task force to update clinical guidelines on how to approach HDGC in 2015 [3]. The case series presented here, from the Cancer Treatment Centers of America in Zion, Illinois, describes the cases and management of three siblings with a mother, sister, and grandparents who had passed away from HDGC or LBC. Being one of the earliest case reports since the updated guidelines, we placed special emphasis in addressing several important points addressed within those guidelines.

## Case presentation

### Case 1

Patient 1 is a 34-year-old Caucasian male with a past medical history of gastroesophageal reflux disease (GERD) and peptic ulcer disease (PUD) who presented to the emergency department of an outside hospital with sudden onset and worsening epigastric pain. A computed tomography (CT) scan of the abdomen showed mild ascites within the pelvic cavity and thickening of the gastric antrum. Transabdominal ultrasound confirmed a small amount of ascites that did not require paracentesis. Esophagogastroduodenoscopy (EGD) revealed a chronic-looking, deep ulcer with radiating folds at the antral region of the lesser curvature of the stomach measuring 1.5 cm in diameter. Biopsy of the specimen revealed poorly differentiated, signet ring cell carcinoma (SRCC) without *Helicobacter pylori* co-infection. Positron emission tomography (PET) scan indicated active disease in the stomach and no evidence of locoregional or distant metastasis.

At this point, the patient presented at our institution for a specialized, second opinion on the management of his malignancy. Endoscopic ultrasound (EUS) and diagnostic laparoscopy with peritoneal washings did not identify nodal involvement or intraperitoneal metastatic disease, respectively, clinically staging the tumor as cT2N0M0. Per NCCN guidelines, the patient underwent three cycles of neoadjuvant chemotherapy with ECX regimen (epirubicin 50 mg/m^2^, cisplatin 60 mg/m^2^, and capecitabine/xeloda 625 mg/m^2^), which were tolerated well by the patient. Re-staging CT scan of the abdomen showed moderate regression of the cancer. Four weeks after completion of the last dose of ETC, the patient underwent total gastrectomy and omentectomy with *Roux-en-Y* esophagojejunostomy and feeding jejunostomy tube (j-tube) placement.

**Fig. 1:**
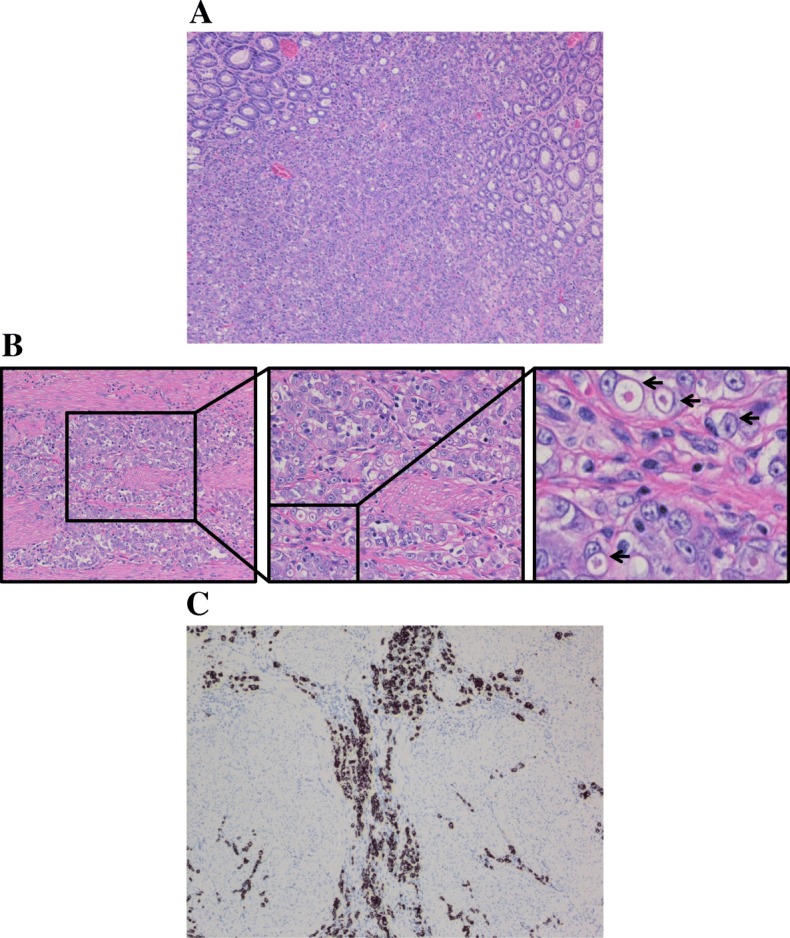
Patient 1 tissue biopsy. **a** Low-power field of hematoxylin and eosin (H&E) staining of gastric mucosa showing disorganized infiltrative sheets of cells invading the lamina propria. **b** Varying degrees of high-power fields demonstrating sheets of signet ring cells (arrows) infiltrating the muscularis mucosa. **c** Immunohistochemistry (IHC) with cytokeratin 7 confirming tumor tissue infiltrating muscularis mucosa

**Fig. 2:**
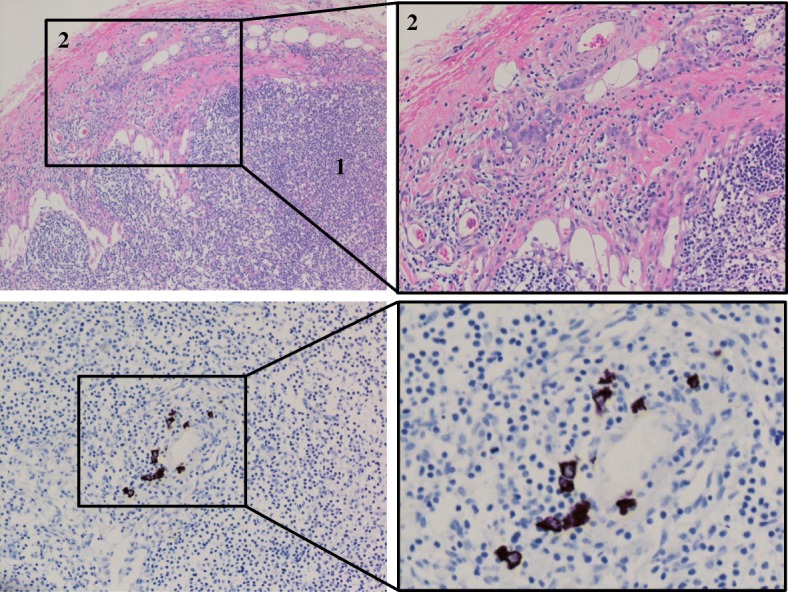
Patient 1 lymph node infiltration. **a** H&E and **b** cytokeratin AE1/AE3 IHC staining of one of the three positive lymph nodes showing disorganized epithelial tissues present and infiltrating the matrix of the lymph node

Pathology of the tissue revealed invasive, poorly differentiated gastric adenocarcinoma with singlet ring cell features that invaded into the muscularis propria and subserosal tissue, but with no evidence of invasion of the visceral peritoneum (T3) (Fig. 1a, b). Presence of malignant tissue was also confirmed with cytokeratin 7 immunostaining (Fig. 1c). The size of the tumor was 2.2 × 2 × 0.6 cm with negative margins, but with 3 of 41 lymph nodes positive for metastatic adenocarcinoma as evidenced by cytokeratin AE1/AE3 immunostaining (N2) (Fig. 2). With no identified distant metastases, the pathologic staging of the tumor was ypT3N2M0, stage IIIA.

The patient recovered post-operatively without complications. Upon resumption of oral and j-tube feedings, the patient was discharged from the hospital and returned to work approximately 1 month later. Six weeks post-surgery, the patient began adjuvant chemotherapy with TCX (epirubicin was substituted for taxotere) for three cycles. Patient is currently under surveillance, and the most recent CT scan of the chest, abdomen, and pelvis identified no active signs of disease. Up to the time of the preparation of this manuscript, the patient continues to follow without clinic and currently displays no evidence of disease.

During the early course of medical workup at our institution, the patient received genetic counseling based on the high incidence of gastric and breast cancer in the family. Specifically, his mother was diagnosed with gastric cancer and died of the disease at the age of 49, while his sister died of the same disease at the age of 25. Further inquiry revealed that the maternal grandfather had also succumbed to what was likely gastric cancer based on the disease course provided by our patient, although a definitive diagnosis was never made. Furthermore, two maternal aunts were diagnosed with breast cancer in their 50s and 60s. The strong prevalence of these two cancers within the patient’s maternal lineage raised suspicion for possible hereditary diffuse gastric carcinoma (HDGC) secondary to a genetic mutation in the *CDH1* gene, particularly based on the recently updated guidelines [3]. Subsequent genetic analysis at our institution confirmed a monoallelic deletion of exons 1–2 of the *CDH1* gene, further corroborating the clinical diagnosis.

### Case 2

Patient 2 is a 32-year-old male and a younger sibling of patient 1. Given the recently identified *CDH1* mutation and HDGC diagnosis in his sibling, patient 2 had a 50% likelihood of being a *CDH1* mutation carrier. Subsequent genetic screening at our institution confirmed that similarly to his older sibling, patient 2 had a monoallelic deletion of exons 1–2 of the *CDH1* gene, predisposing him to the HDGC like several members of his family.

**Fig. 3:**
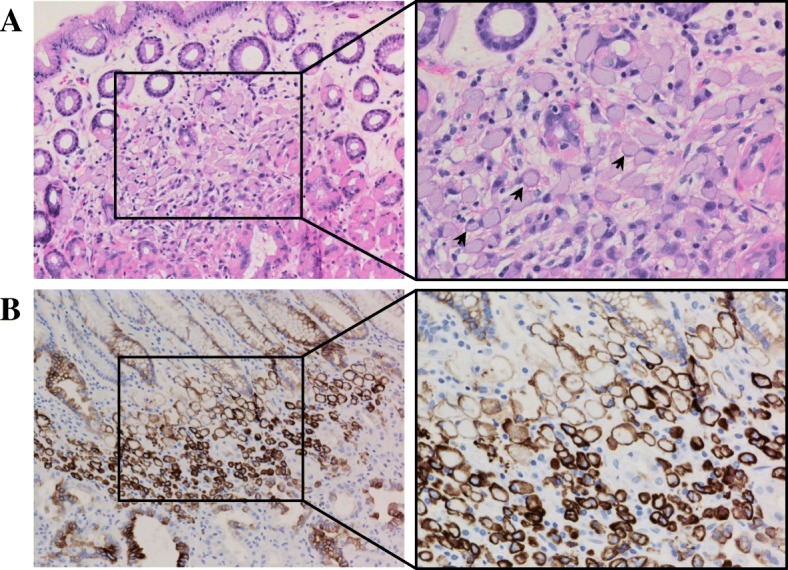
Patient 2 gastric tissue biopsy. **a** High-power field of H&E staining of gastric mucosa with magnification on the right panel showing islet of signet ring cells (arrows) invading the lamina propria. **b** Immunohistochemistry (IHC) with cytokeratin 7 confirming tumor tissue infiltrating the lamina propria

Initial CT scan of the chest, abdomen, and pelvis and EGD biopsy of gastric tissue indicated no sign of active malignancy. However, given the ~ 70% lifetime chance of developing HDGC, the patient was recommended prophylactic gastrectomy despite showing no signs or symptoms of disease. The patient agreed with the recommendation and underwent prophylactic total gastrectomy with *Roux-en-Y* esophagojejunostomy and feeding j-tube placement. Immunohistochemical analysis of gastric and intestinal tissue identified three microscopic foci of signet ring cells in the lamina propria without invasion of the submucosa (Fig. 3a, b), consistent with poorly differentiated adenocarcinoma of the stomach. The rest of the intestinal tract showed no signs of malignancy, and 0 of 30 tested lymph showed positive for metastatic carcinoma. The tumor was pathologically staged as pT1aN0M0.

The patient recovered without complications and was discharged home on post-operative day 7. He returned to the emergency department 5 days later due to diffuse abdominal pain, dark-colored emesis, and no bowel movements for 2 days. Initial CT scan of abdomen and pelvis revealed dilated, gas-filled, small bowel loops. With the presumed diagnoses of ileus vs. partial small bowel obstruction, the patient was re-admitted to the surgical floor for further management. Subsequent tests were unremarkable except for elevated amylase of 286 U/L and lipase of 1153 U/L, suggesting pancreatitis as a more likely source for his abdominal pain. The patient was managed per pancreatitis protocol and recovered well. He was subsequently discharged from our institution and continues to do well without evidence of disease at the time of this manuscript.

### Case 3

**Fig. 4:**
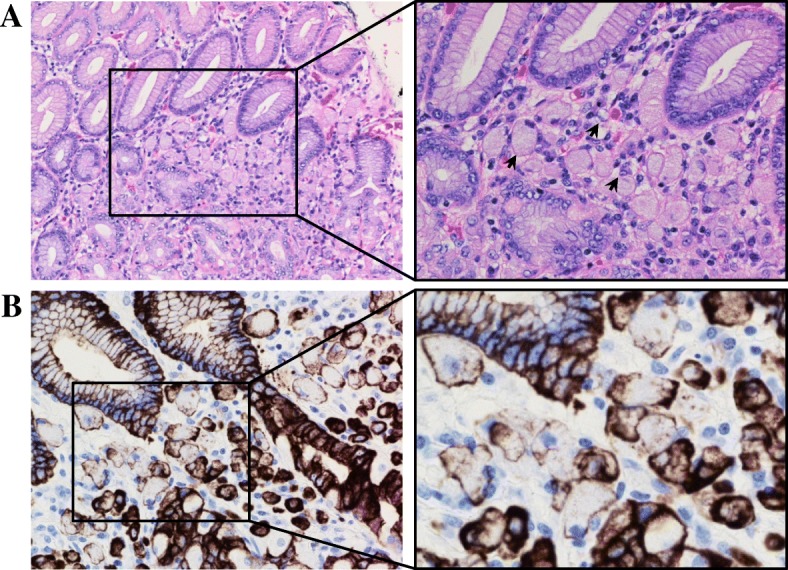
Patient 3 gastric tissue biopsy. **a** High-power field of H&E staining of gastric mucosa with magnification on the right panel showing islet of signet ring cells (arrows) invading the lamina propria. **b** Immunohistochemistry (IHC) with cytokeratin 7 confirming tumor tissue infiltrating the lamina propria

Patient 3 is a 23-year-old female and youngest sibling of the two aforementioned patients. Her past medical history was relevant for generalized anxiety disorder and gastritis, while family history was relevant for an offspring with cleft lip, another condition associated with *CDH1* mutations. Her genetic screening revealed presence of the same genetic mutation that afflicted her older siblings, namely, monoallelic deletion of exons 1–2 of the *CDH1* gene. Initial CT scan of the chest, abdomen, and pelvis and EGD biopsy were unremarkable. Similarly to her older brother, she agreed to undergo prophylactic total gastrectomy with *Roux-en-Y* esophagojejunostomy and j-tube placement despite exhibiting no active signs or symptoms of disease. Immunohistochemical analysis of gastric and intestinal tissue revealed multiple microscopic signet ring cell foci varying in size, with the largest measuring 1 mm at its largest diameter (Fig. 4a, b). The tumor was confined to the lamina propria without evidence of invasion into the submucosa. All 23 tested lymph nodes tested negative for metastatic disease, and no other organ revealed signs of malignancy, staging the tumor as pT1aN0M0.

The patient recovered without major complications and was discharged on post-operative day 7 after abdominal pain and nutrition were adequately managed. Furthermore, the patient was offered prophylactic bilateral mastectomy given the increased incidence of LBC in women with *CDH1* mutations. Initially, the patient refused the procedure citing the desire to breastfeed her future children and the low incidence of the disease prior to the age of 30. However, she subsequently consented to the procedure and underwent successful prophylactic bilateral mastectomy. During her outpatient recovery, patient 3 developed symptomatic gallstones. She re-admitted for a third time to undergo elective cholecystectomy and recovered without complications. She continues to follow-up with our clinic and currently displays no evidence of disease.

## Discussion

Although gastric cancer (GC) is a leading cause of cancer mortality worldwide, hereditary diffuse gastric cancer (HDGC) is relatively rare. As a consequence, best management options are based mostly on conclusions drawn from small case series articles and retrospective analyses and are therefore still not entirely agreed upon. In 2015, a multidisciplinary workshop revised the criteria for diagnosing HDGC mainly based on a recent large *CDH1* sequencing study in patients with HDGC [3, 4]. Part of the revisions involved updating the criteria for HDGC diagnosis. New guidelines recommend establishing a diagnosing of HDGC if the one or more of the following are present: (i) two diagnoses of GC in a family, regardless of age, with at least one confirmed as diffuse GC; (ii) one diagnosis of DGC in someone younger than 40; or (iii) personal or family history of DGC and LBC, with at least one before the age of 50 [3]. In this report, we presented a case series of three siblings who fulfilled all three HDGC diagnostic criteria, making this one of the earliest clinical reports since the new guidelines were implemented. We closely followed the new guidelines and address below some of the salient issues that arose during the management of these patients.

Hansford et al. undertook the ambitious effort of genetically sequencing the blood of 183 patients with the clinical diagnosis of HDGC [4]. To date, that is the largest study investigating *CDH1* mutations in the context of HDGC and provides invaluable yet difficult data to discuss with patients. Specifically, they found that men harboring germline *CDH1* mutations have a 70% chance of developing HDGC by the age of 80, while women have a 56% of developing the same.

While to many patients the risks inherent in these numbers are unacceptable and immediate prophylactic gastrectomy is an obvious decision regardless of age, for others, whether and when to have such a life-altering procedure can be a particularly sensitive topic. This decision is made difficult by several conflicting factors. On the one hand, prophylactic gastrectomy is curative (i.e., it reduces the risk of developing HDGC to virtually 0%); this contrasts drastically with the fact that patients who develop diffuse GC have a dismal 5-year overall survival rate of 10–20% [6]. On the other hand, prophylactic gastrectomy itself comes with numerous life-altering implications and risks such as infections, dumping syndrome, and a post-operative mortality rate of approximately 1% [7, 8]. Indeed, two of the patients’ relatives who had tested positive for *CDH1* at a later date and were treated at outside institutions suffered major complications post-operatively, with an aunt suffering an anastomotic stricture post-gastrectomy, while another dying from pulmonary embolism complications. Additionally, these patients are also afflicted with long-term alteration of their ability to digest and absorb nutrients, often resulting in drastic weight loss and permanent GI disturbance [9, 10]. This can have both physical as well as psychosocial implications for afflicted patients.

These conflicting interests became evident in our case series. Patients 2 and 3 were willing to undergo immediate prophylactic gastrectomy upon positive genetic testing as they believed the risk of developing diffuse GC far outweighed the risks and side effects of total gastrectomy. A fourth sibling, who was not included in the study, refused gastrectomy at this time because he believed the opposite: the risk of developing GC at this time was not as consequential for him as the outcome of total gastrectomy. Deciding the ideal time to undergo prophylactic gastrectomy can be very challenging even with statistical models in hand. Even within this family, the time of onset of GC varied dramatically, with patient 1 at 34, the mother at 49, and another sister at 25. It is important to emphasize that patients who forego prophylactic gastrectomy should undergo annual screening with EGD and biopsies [3, 4]. However, as evidenced by the fact that screening EGD biopsies failed to identify the carcinoma in situ in both cases 2 and 3, annual EGD should not be offered as an equivalent alternative to prophylactic gastrectomy, but rather it should be reserved only for patients who refuse or delay prophylactic treatment.

It is also important to note that patient 2 suffered significant emotional and physical distress before, during, and after the procedure. In addition to the brief complication of pancreatitis after surgery, he continuously expressed anxiety regarding his muscle weight loss and difficulty with oral food intake. To help him through the process, we followed the updated guidelines, planned and executed numerous scheduled phone calls with our nutritionist, psychologist, and genetic counselor to help him through the early post-operative stages, when most of these symptoms were prevalent. After months of close follow-up, the patient has fully recovered on a nutritional perspective and resumed arm-wrestling, his personal hobby.

Although patient 3 underwent prophylactic gastrectomy, she initially decided not to undergo prophylactic bilateral mastectomy, which would drastically reduce her risk of developing LBC in the future. Citing her desire to breastfeed her current and potential future children, she decided to proceed with annual screening mammography and MRI starting at the age of 30, as per the new guidelines [3, 4]. However, upon further counseling with family members, our geneticist, social worker, and other healthcare professionals, she reversed her original decision and decided to proceed with prophylactic mastectomy instead. This case further demonstrates that determining when a patient should undergo a prophylactic procedure should be individualized and based on patients’ goals, especially for women of childbearing age. Even when tailored to a specific patient, the treatment plan should be fluid, and the healthcare team should be ready to accommodate patients’ changing preferences.

Since over 92% of prophylactic gastrectomies in *CDH1* mutated patients with family history of HDGC will reveal SRCC on pathologic examination, a recent study suggested presenting the total gastrectomy procedure to patients as a *curative*, rather than a prophylactic option [11, 12]. This, as hypothesized, would increase patients’ inclination to undergo the procedure, as patients are inclined to get curative rather than preventative treatments. Consisted with these data, both our asymptomatic patients (patients 2 and 3) showed evidence of SRCC despite having no evidence of disease on imaging and endoscopic sampling. It could be possible that the fourth sibling may have decided to undergo total gastrectomy if the procedure was offered as a curative, rather than as a prophylactic procedure, and that his histologic findings would possibly reveal SRCC. However, we believe that such semantics should not play a major role in a patient’s decision to undergo such an important surgical procedure. Rather, the authors believe that regardless of the terminology used, the patient should be offered all the facts at our disposal about the risks and benefits of undergoing a certain procedure and allow the patient to make the decision that best suits one’s life philosophy and goals.

Although our article centers on a case series with HDGC secondary to a germline *CDH1* mutation, it is important to note that a diagnosis of HDGC is made based on clinical and family history and is not ruled out by a negative *CDH1* genetic testing results. In fact, the Hansford et al. study found that only 34 out of 183, or not even 20% of patients with clinical diagnosis of HDGC, harbored *CDH1* mutations [4]. While this number is lower than the 40% that is historically reported [13–16], it does underscore the fact that absence of a readily identifiable mutation in patients with strong clinical and family history suspicious for HDGC does not rule out the presence of the condition and that further testing, such as sequencing other genes, can and should be pursued. The same study by Hansford et al. identified *CTNNA1*, a gene that codes for α-catenin, as another culprit in HDGC, and that should be screened in patients where clinical suspicion is high but *CDH1* genetic testing is unremarkable [4].

Conversely, while the presence of a germline *CDH1* mutation strongly increases the clinical suspicion of HDGC, particularly in patients with family history of gastric or lobular cancer, not all *CDH1* mutations are pathogenic or cause HDGC. A study from France sequenced 165 individuals at random and identified 18 germline *CDH1* mutations [17]. Of these, only 11 fulfilled the new criteria of HDGC, suggesting that a mere germline *CDH1* mutation is not sufficient to attribute a diagnosis of HDGC in a family.

## Conclusions

Gastric cancer continues to be a leading cause of cancer-related mortality worldwide, and HDGC continues to be measurable and potentially a preventable contributor to these deaths. To our knowledge, here we presented one of the earliest case series on *CDH1*-mediated HDGC since the implementation of the new HDGC guidelines. We noted that updated diagnostic guidelines appear to adequately diagnose the disease, as our patients’ family fulfilled all three criteria at once. Interestingly, despite recent knowledge about the disease and clinical recommendations, the best course of action in each case continues to remain a sensitive subject and should be individualized for each patient. We noted that a strong multidisciplinary support group including geneticists, clinical and surgical oncologists, nutritionist, psychologist, and social workers is key in successfully carrying these patients through this challenging journey. Lastly, although presence of *CDH1* mutation in a family with clear history of DGC and LBC, it is important to remember that additional mutations can also cause HDGC.
